# MetRxn: a knowledgebase of metabolites and reactions spanning metabolic models and databases

**DOI:** 10.1186/1471-2105-13-6

**Published:** 2012-01-10

**Authors:** Akhil Kumar, Patrick F Suthers, Costas D Maranas

**Affiliations:** 1 Department of Computer Science, The Pennsylvania State University, University Park, PA 16802, USA; 2 Department of Chemical Engineering, The Pennsylvania State University, University Park, PA 16802, USA

## Abstract

**Background:**

Increasingly, metabolite and reaction information is organized in the form of genome-scale metabolic reconstructions that describe the reaction stoichiometry, directionality, and gene to protein to reaction associations. A key bottleneck in the pace of reconstruction of new, high-quality metabolic models is the inability to directly make use of metabolite/reaction information from biological databases or other models due to incompatibilities in content representation (i.e., metabolites with multiple names across databases and models), stoichiometric errors such as elemental or charge imbalances, and incomplete atomistic detail (e.g., use of generic R-group or non-explicit specification of stereo-specificity).

**Description:**

MetRxn is a knowledgebase that includes standardized metabolite and reaction descriptions by integrating information from BRENDA, KEGG, MetaCyc, Reactome.org and 44 metabolic models into a single unified data set. All metabolite entries have matched synonyms, resolved protonation states, and are linked to unique structures. All reaction entries are elementally and charge balanced. This is accomplished through the use of a workflow of lexicographic, phonetic, and structural comparison algorithms. MetRxn allows for the download of standardized versions of existing genome-scale metabolic models and the use of metabolic information for the rapid reconstruction of new ones.

**Conclusions:**

The standardization in description allows for the direct comparison of the metabolite and reaction content between metabolic models and databases and the exhaustive prospecting of pathways for biotechnological production. This ever-growing dataset currently consists of over 76,000 metabolites participating in more than 72,000 reactions (including unresolved entries). MetRxn is hosted on a web-based platform that uses relational database models (MySQL).

## Background

**Figure 1:**
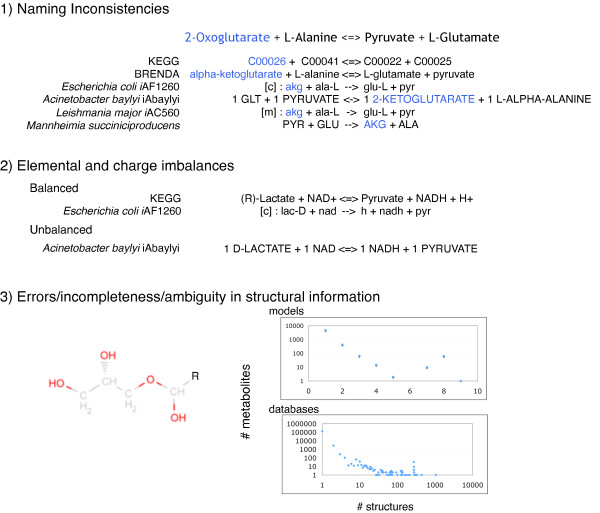
**Typical incompatibilities and inconsistencies in genome-scale models and databases**. Roadblocks to using genome-scale models and databases include ambiguities and differences in naming conventions, lack of balanced reactions, and incompleteness of structural information.

The ever accelerating pace of DNA sequencing and annotation information generation [1] is spearheading the global inventorying of metabolic functions across all kingdoms of life. Increasingly, metabolite and reaction information is organized in the form of community [2], organism, or even tissue-specific genome-scale metabolic reconstructions. These reconstructions account for reaction stoichiometry and directionality, gene to protein to reaction associations, organelle reaction localization, transporter information, transcriptional regulation and biomass composition. Already over 75 genome-scale models are in place for eukaryotic, prokaryotic and archaeal species [3] and are becoming indispensable for computationally driving engineering interventions in microbial strains for targeted overproductions [4-7], elucidating the organizing principles of metabolism [8-11] and even pinpointing drug targets [12,13]. A key bottleneck in the pace of reconstruction of new high quality metabolic models is our inability to directly make use of metabolite/reaction information from biological databases [14] (e.g., BRENDA [15], KEGG [16], MetaCyc, EcoCyc, BioCyc [17], BKM-react [18], UM-BBD [19], Reactome.org, Rhea, PubChem, ChEBI etc.) or other models [20] due to incompatibilities of representation, duplications and errors, as illustrated in Figure 1.

A major impediment is the presence of metabolites with multiple names across databases and models, and in some cases within the same resource, which significantly slows down the pooling of information from multiple sources. Therefore, the almost unavoidable inclusion of multiple replicates of the same metabolite can lead to missed opportunities to reveal (synthetic) lethal gene deletions, repair network gaps and quantify metabolic flows. Moreover, most data sources inadvertently include some reactions that may be stoichiometrically inconsistent [21] and/or elementally/charge unbalanced [22,23], which can adversely affect the prediction quality of the resulting models if used directly. Finally, a large number of metabolites in reactions are partly specified with respect to structural information and may contain generic side groups (e.g., alkyl groups -R), varying degree of a repeat unit participation in oligomers, or even just compound class identification such as "an amino acid" or "electron acceptor". Over 3% of all metabolites and 8% of all reactions in the aforementioned databases and models exhibit one or more of these problems.

There have already been a number of efforts aimed at addressing some of these limitations. The Rhea database, hosted by the European Bioinformatics Institute, aggregates reaction data primarily from IntEnz [24] and ENZYME [25], whereas Reactome.org is a collection of reactions primarily focused on human metabolism [26,27]. Even though they crosslink their data to one or more popular databases such as KEGG, ChEBI, NCBI, Ensembl, Uniprot, etc., both retain their own representation formats. More recently, the BKM-react database is a non-redundant biochemical reaction database containing known enzyme-catalyzed reactions compiled from BRENDA, KEGG, and MetaCyc [18]. The BKM-react database currently contains 20,358 reactions. Additionally, the contents of five frequently used human metabolic pathway databases have been compared [28]. An important step forward for models was the BiGG database, which includes seven genome-scale models from the Palsson group in a consistent nomenclature and exportable in SBML format [29-31]. Research towards integrating genome-scale metabolic models with large databases has so far been even more limited. Notable exceptions include the partial reconciliation of the latest *E. coli *genome scale model *i*AF1260 with EcoCyc [32] and the aggregation of data from the *Arabidopsis thaliana *database and KEGG for generating genome-scale models [33] in a semi-automated fashion. Additionally, ReMatch integrates some metabolic models, although its primary focus is on carbon mappings for metabolic flux analysis [34]. Also, many metabolic models retain the KEGG identifiers of metabolites and reactions extracted during their construction [35,36]. An important recent development is the web resource Model SEED that can generate draft genome-scale metabolic models drawing from an internal database that integrates KEGG with 13 genome scale models (including six of the models in the BiGG database) [37]. All of the reactions in Model SEED and BiGG are charge and elementally balanced.

In this paper, we describe the development and highlight applications of the web-based resource MetRxn that integrates, using internally consistent descriptions, metabolite and reaction information from 8 databases and 44 metabolic models. The MetRxn knowledgebase (as of October 2011) contains over 76,000 metabolites and 72,000 reactions (including unresolved entries) that are charge and elementally balanced. By conforming to standardized metabolite and reaction descriptions, MetRxn enables users to efficiently perform queries and comparisons across models and/or databases. For example, common metabolites and/or reactions between models and databases can rapidly be generated along with connected paths that link source to target metabolites. MetRxn supports export of models in SBML format. New models are being added as they are published or made available to us. It is available as a web-based resource at http://metrxn.che.psu.edu.

## Construction and Content

### MetRxn construction

**Figure 2:**
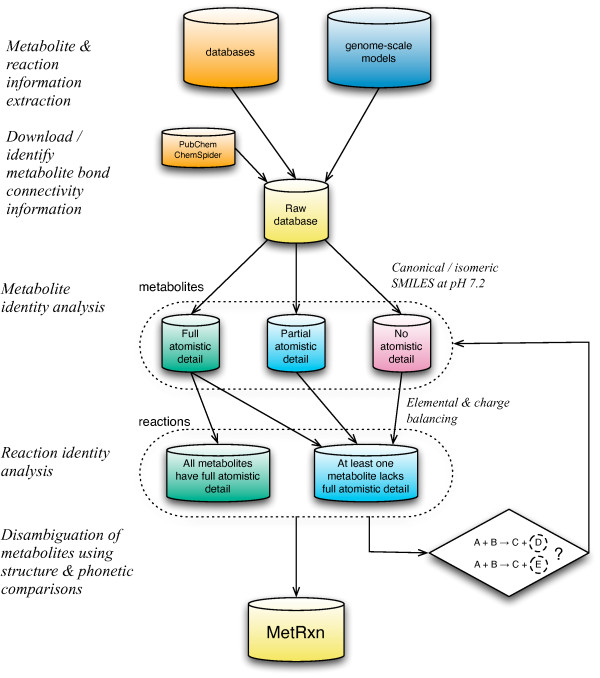
**Flowchart outlining the construction of MetRxn**. After download of primary sources of data from databases and models, we integrated metabolite and reaction data, followed by calculation and reconciliation of structural information. By identifying overlaps between metabolite and reaction information, we generated elemental and charge balancing of reactions. The procedure for developing MetRxn was iterative with subsequent passes making use of previous associations to resolve remaining ambiguities.

The construction of MetRxn largely followed the following steps, as illustrated in Figure 2: 1) download of primary sources of data from databases and models, 2) integration of metabolite and reaction data, 3) calculation and reconciliation of structural information, 4) identification of overlaps between metabolite and reaction information, 5) elemental and charge balancing of reactions, 6) successive resolution of remaining ambiguities in description.

#### Step 1: Source data acquisition

Metabolite and reaction data was downloaded from BRENDA, KEGG, BioCyc, BKM-react and other databases using a variety of methods based on protocols such as SOAP, FTP and HTTP. We preprocessed the data into flat files that were subsequently imported into the knowledgebase. All original information pertaining to metabolite name, abbreviations, metabolite geometry, related reactions, catalyzing enzyme and organism name, gene-protein-reaction associations, and compartmentalization was retained. For all 44 initial genome-scale models listed, the online information from the corresponding publications was also imported. The source codes for all parsers used in Step 1 are available on the MetRxn website.

#### Step 2: Source data parsing

The "raw data" from both databases and models was unified using standard SQL scripts on a MySQL server. The description schema for metabolites includes source, name, abbreviations used in the source, chemical formula, and geometry. The schema for reactions accounts for source, name, reaction string (reactants and products), organism designation, associated enzymes and genes, EC number, compartment, reversibility/direction, and pathway information. Once a source has been imported into the MySQL server, a data source-specific dictionary is created to map metabolite abbreviations onto names/synonyms and structures and metabolites to reactions.

#### Step 3: Metabolite charge and structural analysis

We used Marvin (Chemaxon) to analyze all 218,122 raw metabolite entries containing structural information (out of a total of 322,936, including BRENDA entries). Inconsistencies were found in 12,965 entries typically due to wrong atom connectivity, valence, bond length or stereo chemical information, which were corrected using APIs available in Marvin. A final corrected version of the metabolite geometries was calculated at a fixed pH of 7.2 and converted into standard Isomeric SMILES format. The structure/formula used corresponded to the major microspecies found during the charge calculation, which effectively rounds the charge to an integer value in accordance with previous model construction conventions. This format includes both chiral and stereo information, as it allows specification of molecular configuration [38-40]. Metabolites were also annotated with Canonical SMILES using the OpenBabel Interface from Chemspider. The canonical representation encodes only atom-atom connectivity while ignoring all conformers for a metabolite. Using bond connectivity information from the primary sources and resources such as PubChem and ChemSpider we used Canonical SMILES [41,42] to resolve the identity of 34,984 metabolites and 32,311 reactions. Another 6,100 metabolites and 11,401 reactions involved, in various degrees, lack of full atomistic detail in their description (e.g., use an R or × as side-chains, are generic compounds like "amino acid" or "electron acceptor"). Over 25,000 duplicate metabolites and 27,000 reaction entries were identified and consolidated within the database. The metabolites and reactions present in the resolved repository were further classified with respect to the completeness of atomistic detail in their description.

#### Step 4: Metabolite synonyms and initial reaction reconciliation

Raw metabolite entries were assigned to Isomeric SMILES representations whenever possible. If insufficient structural information was available for a downloaded raw metabolite then it was assigned temporarily with the Canonical SMILES and revisited during the reaction reconciliation. Canonical SMILES retain atom connectivity but not stereo-specificity and are used as the basic metabolite topology descriptors as many metabolic models lack stereo-specificity information. After generating the initial metabolite associations, we identified reaction overlaps using the reaction synonyms and reaction strings along with the metabolite SMILES representations. Directionality and cofactor usage were temporarily ignored. During this step, reactions were flagged as single-compartment or two-compartment (i.e., transport reactions). MetRxn internally retains the original compartment designations, but currently only displays these simplified compartment designations. In analogy to metabolites, reactions were grouped into families that shared participants but in the source data sets occurred in different compartments or differed only in protonation.

#### Step 5: Reaction charge and elemental balancing

Once metabolites were assigned correct elemental composition and protonation states, reactions were charge and elementally balanced. To this end, for charge balancing we relied on a linear programming representation that minimizes the difference in the sum of the charge of the reactants and the sum of the charge on the products. The complete formulation is provided in the documentation at MetRxn.

#### Step 6: Iterative reaction reconciliation

Reactions with one (or more) unresolved reactants and/or products were string compared against the entire resolved collection of reactions. This step was successively executed as newly resolved metabolites and reactions could enable the resolution of previously unresolved ones. After the first pass 164 metabolites were resolved, while subsequent passes (up to 18 for some models) helped resolved a total of 8,720 entries. Reactions with significant (but not complete) overlapping sets of reactants/products are additionally sent to the curator GUI including phonetic information. Briefly, the phonetic tokens of synonyms with known structures were compared against the ones without any associated structure. The algorithm suppresses keywords/tokens depicting stereo information such as *cis, trans*, L-, D-, alpha, beta, gamma, and numerical entries because they change the phonetic signature of the synonym under investigation. In addition, the algorithm ignores non-chemistry related words (e.g., use, for, experiment) that are found in some metabolite names. Certain tokens such as "-ic acid" and "-ate" are treated as equivalent. PubChem and Chemspider sources were accessed through the GUI so that the curator gets as much information as possible to identify the data correctly. Phonetic matches provided clues for resolving over 159 metabolites. The iterative application of string and phonetic comparison algorithms resolved as many as 8,879 metabolites after 18 rounds of reconciliation.

Upon completion of this workflow, all genome-scale models are reformatted into a computations-ready form and Flux Balance Analysis [43] is performed on both the source model and the standardized model in MetRxn to ascertain the ability of the model to produce biomass before and after standardization. We performed the calculations using GAMS version 12.6. MetRxn is accessible through a web interface that indirectly generates MySQL queries. In order to facilitate analysis and use of the data, a number of tools are provided as part of MetRxn.

### Data export and display

MetRxn supports a number of export capabilities. In general, any list that is displayed contains live links to the metabolite or reaction entities. These lists can consist of an entire model, data from a comparison, or query results. All items can be exported to SMBL format. In addition, the public MySQL database will be made available upon request. Because of licensing limitations, the BRENDA database cannot be exported and is not part of the public MySQL database. However, we plan to provide Java source code that allows for the integration of a local copy of the public MySQL database with the BRENDA database (provided upon request).

### Source comparisons and visualization

In addition to listing the content (number of metabolites, reactions, etc.) of the selected data source(s), MetRxn contains tools for comparing two or more models and visualizing the results. These associations can be for metabolites or reactions. During these comparisons compartment information and reversibility are suppressed. Comparison tables are generated by comparing the associations between the selected data source(s) using the canonical structures.

### MetRxn Scope

**Figure 3:**
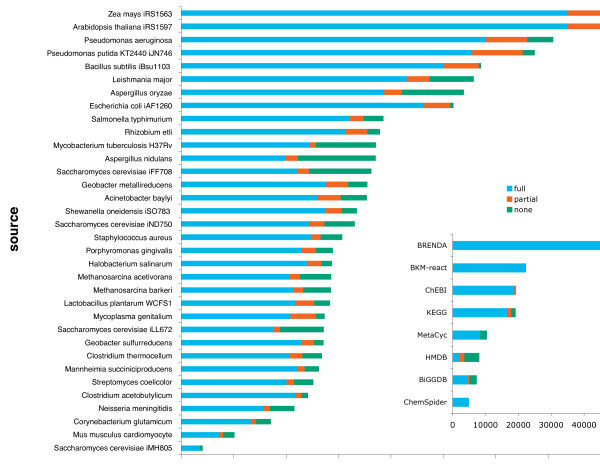
**Various levels of structural information was available for models (main) and databases (inset)**. For every model, the majority of metabolites had full atomistic detail (blue). The smaller number of metabolites with partial atomistic detail (orange) such as genetic side chains, or with no atomistic detail (green) such as gene products, participated in few reactions.

An initial repository of reaction (i.e., 154,399) and metabolite (i.e., 322,936) entries were downloaded from 8 databases and 44 genome-scale metabolic models. We compiled a non-redundant list of 42,540 metabolites and 35,474 reactions (after consolidating duplicate entries) containing full atomistic and bond connectivity detail. Another 6,100 metabolites and 11,401 reactions have partial atomistic detail typically containing generic side-chains (R) and/or an unspecified number of polymer repeat units. Finally, 5,436 metabolites in metabolic models and 8,000 metabolites in databases are retained with no atomistic detail. In some cases lack of atomistic detail reflects complete lack of identity specificity (e.g., electron donor) whereas in other cases even though the chemical species is fully defined, atomistic level description is not warranted (e.g., gene product of dsbC protein disulfide isomerase II (reduced)). Figure 3 shows the distribution of metabolite resolution across models and databases in MetRxn. In general, metabolites without fully-specified structures tend to participate in a relatively small number of reactions.

The workflow followed in the creation of the MetRxn knowledgebase identified a number of inconsistencies. For instance, the same metabolite name may map to molecules with different numbers of repeat units (e.g., lecithin) or completely different structures (e.g., AMP could refer to either adenosine monosphate or ampicillin). Notably, even for the most well-curated metabolic model, *E. coli i*AF1260 [32], we found minor errors or omissions (a total of 17) arising from inconsistencies or incompleteness of representation in the data culled from other sources. For example, the metabolite abbreviation arbtn-fe3 was mistakenly associated with the KEGG ID and structure of aerobactin instead of ferric-aerobactin. The number of inconsistencies is dramatically increased for less-curated metabolic models. We used a variety of procedures to disambiguate the identity of metabolites lacking structural information ranging from reaction matching to phonetic searches. For example, in the *Corynebacterium glutamicum *model [44], 7,8-aminopelargonic acid (DAPA) has no associated structural information. Reaction matching found the same reaction in the *E. coli i*AF1260 model.

C. glutamicumDAPA+ ATP+ CO2⇔ DTBIOTIN+ ADP+ PI

$$i\text{AF126}0\left[\text{c}\right]:\text{ atp }+\text{ co2 }+\text{ dann }\to \text{ adp }+\text{ dtbt }+\;\left(\text{3}\right)\text{ h }+\text{ pi}$$

which implies that 7,8-aminopelargonic acid (DAPA) is identical to 7,8-Diaminononanoate (dann). Examination of pelargonic acid and nonanoate reveals that they were indeed known synonyms. In many cases, we were also able to assign stereo-specific information to metabolite entries in models (e.g., stipulate the L-lysine isomer for lysine). We made use of an iterative approach that allowed us to map structures from models with explicit links to structures (e.g. to KEGG or CAS numbers) to models that only provided metabolite names. Furthermore, by using a phonetic algorithm that uses tokens for equivalent strings in metabolite names (e.g., '-ic acid' and '-ate' are equivalent) we were able to resolve more than an additional 159 metabolites. For example, phonetic searches flagged cis-4-coumarate and COUMARATE in the *Acinetobacter baylyi *model [45] as potentially identical compounds. Additional checks revealed that indeed both metabolites should map to the same structure. A more complex matching example involved 1-(5'-Phosphoribosyl)-4-(N-succinocarboxamide)-5-aminoimidazole from the *Bacillus subtilis *model [46] and 1-(5'-Phosphoribosyl)-5-amino-4-(N-succinocarboxamide)-imidazole from the *Aspergillus nidulans *model [47] . We note that the phonetic algorithm only makes suggestions and orders the possible matches for the curator. Next, we detail three examples that provide an insight into the type of tasks that MetRxn can facilitate.

## Utility and Discussion

### 1. Charge and elementally balanced metabolic models

The standardized description of metabolites and balanced reactions afforded by MetRxn enables the expedient repair of existing models for metabolite naming inconsistencies and reaction balancing errors. Here we highlight one such metabolic model repair for *Acinetobacter baylyi i*Abaylyi^v4 ^[45]. We identified that 189 out of 880 reactions are not elementally or charge balanced. Most of the reactions with charge balance errors involved a missed proton in reactions involving cofactor pairs such as NAD/NADH. For example, a proton had to be added to the reactants side in the reaction (R, R)-Butanediol-dehydrogenase in which butanediol reacts with NAD to form acetoin. In addition, the stoichiometric coefficient of water in GTP cyclohydrolase I was erroneously set at -2 which resulted in an imbalance in oxygen atoms. The re-balancing analysis changed the coefficient to -1 (as listed in BRENDA) and added a proton to the list of reactants (absent from BRENDA) in order to also balance charges.

We performed flux balance analysis (FBA) on both the published and MetRxn-based rebalanced version of the *Acinetobacter baylyi *model using the uptake constraints listed in [45] to assess the effect of re-balancing reaction entries on FBA results. We found that the maximum biomass using the glucose/ammonia uptake environment decreased by 9% primarily due to the increased energetic costs associated with maintaining the proton gradient. This result demonstrates the significant effect that lack of reaction balancing may cause in FBA calculations. Overall, we found that nearly two-thirds of the models had at least one unbalanced reaction, with over 2,400 entities across all models that were either charge or elementally imbalanced. Frequently, the same reaction was imbalanced in multiple models (each occurrence was counted separately).

### 2. Contrasting existing metabolic models

At the onset of creating MetRxn, we conducted a brief preliminary study to quantify the extent/severity of naming inconsistencies by contrasting the reaction information contained in an initial collection of 34 of the most popular genome-scale models spanning 21 bacterial, 10 eukaryotic and three archaeal organisms. Across all branches of life, most metabolic processes are largely conserved (e.g., glycolysis, pentose phosphate pathway, amino acid biosynthesis, etc.) therefore we expected to uncover a large core of common reactions shared by all models. Surprisingly, we found that only three reactions (i.e., phosphoglycerate mutase, phosphoglycerate kinase, and CO_2 _transport) were directly recognized as common across those 34 models using a simple string match comparison. Even when examining models for only a few bacterial organisms (*Bacillus subtilis, Escherichia coli, Mycobacterium tuberculosis, Mycoplasma genitalium*, and *Salmonella *Typhimurium) simple text searches recognized only 40 common reactions (out of a possible 262, which is the size of the *M. genitalium *model). The reason for this glaring inconsistency is that differing metabolite naming conventions, compartment designations, stoichiometric ratios, reversibility, and water/proton balancing issues prevents the automated recognition of genuinely shared reactions across models. Using the glucose-6-phosphate dehydrogenase reaction as a representative example, Table 1 reveals some of the reasons for failing to automatically recognize common reactions across selected models [13,32,35,36,47-64]. As many as nine different representations of the same reaction exist due to incomplete elemental and charge balancing, alternate cofactor usage among different organisms, and lack of universal metabolite naming conventions. We have found that this level of discord between models is representative for most metabolic reactions. This lack of consistency renders direct pathway comparisons across models meaningless and the aggregation of reaction information from multiple models precarious. This deficiency motivated the development of MetRxn. Given standardization in metabolite naming and elementally/charge balanced reaction entries MetRxn allows for the identification of shared reactions as well as differences between any two metabolic models (assuming that all the metabolites in the compared reaction entries have full atomistic information). When making the comparison of those same metabolic models, MetRxn found an additional 15 reactions in common (for a total of 55 -- a 38% increase) and that 142 reactions are shared by *B. subtilis, E. coli *and *Salmonella *Typhimurium.

**Table 1:** Representation of glucose-6-phosphate dehydrogenase in selected metabolic models

Reaction	Model/Citation
[c]: g6p + nadp < = = > 6pgl + h + nadph	*Escherichia coli *[32]; *Lactobacillus plantarum *[48]; *Pseudomonas aeruginosa *[49]; *Staphylococcus aureus *[50]

[c]: g6p + nadp -- > 6pgl + h + nadph	*Bacillus subtillis *[51]; *Mycobacterium tuberculosis *[13]; *Pseudomonas putida *[52]; *Rhizobium etli *[53]; *Saccharomyces cerevisiae *[54]

[c]g6p + nadp < = = > 6pgl + h + nadph	*Saccharomyces cerevisiae *[55]; *Escherichia coli *[56]

[c]: f420-2 + g6p -- > 6pgl + f420-2h2	*Methanosarcina barkeri *[57]

G6P + NADP < - > D6PGL + NADPH	*Escherichia coli *[58]; *Mus musculus *[59]; *Saccharomyces cerevisiae *[60,61]

G6P + NADP - > D6PGL + NADPH	*Aspergillus nidulans *[47]; *Mannheimia succiniciproducens *[62]; *Streptomyces coelicolor *[63]

G6P + NAD - > D6PGL + NADH	*Helicobacter pylori *[64]

C01172 + C00006 = C01236 + C00005 + C00080	GSM mouse [35]

C00092 + C00006 < = > C01236 + C00005 + C00080	*Halobacterium salinarum *[36]

**Figure 4:**
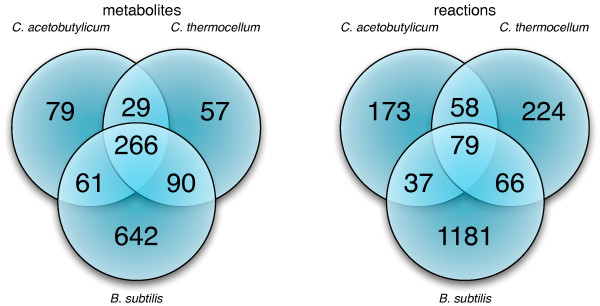
**Comparison of metabolite and reaction overlaps for *C. acetobutylicum *and *C. thermocellum*, and *B. subtilis***. Although the two Clostridium organisms are same genus, the models of these two species had significant numbers of unique metabolites (left) and reactions (right), and comparisons revealed that there was more similarity in metabolite usage with a model of *B. subtilis *than with each other. In part, these overlaps were driven by the explicit accounting for charged tRNA species in *C. thermocellum *and *B. subtilis *models, which was also reflected in the reaction overlaps through reactions involving these metabolites.

The Web interface of MetRxn allows for any number of models to be simultaneously compared. As a demonstration of this capability we selected to contrast the metabolic content of two clostridia models: *Clostridium acetobutylicum *[65] and *Clostridium thermocellum *[66]. Figure 4 shows the results in the form of a Venn diagram. Some of the differences between the clostridia species are not surprising arising due to their differing lifestyles (*C. acetobutylicum *contains solventogenesis pathways and a CoB12 pathway, whereas *C. thermocellum *contains cellulosome reactions). However, we found many differences that appear to reflect different conventions adopted when the two models were generated rather than genuine differences in metabolism. In particular, in the *C. thermocellum *model [66] charged/uncharged tRNA metabolites are explicitly tracked whereas they are not included in the *C. acetobutylicum *model [65]. Surprisingly, both clostridia models are more similar, at the metabolite level, to the *Bacillus subtilis *iBsu1103 model [46] rather than to each other (see Figure 4). Charged/uncharged tRNA metabolites account for most of the increased overlap between *C. thermocellum *and *B. subtilis*. Most of the reaction overlaps are in the amino acids biosynthesis pathways, carbohydrate metabolism, and nucleoside metabolism. It is important to note that 48 reactions in *C. acetobutylicum*, 67 reactions in *C. thermocellum*, and 120 reactions in *B. subtilis *lack full atomistic information (see Figure 3) and thus were excluded from any comparisons. It is possible that additional shared reactions between the two models can be deduced by further examining comparisons between not fully structurally specified metabolite entries. The string/phonetic comparison algorithms described under Step 6 along with assisted curation could be adapted for this task.

### 3. Using MetRxn to Bio-Prospect for Novel Production Routes

A "Grand Challenge" in biotechnological production is the identification of novel production routes that allow for the conversion of inexpensive resources (e.g., various sugars) into useful products (e.g., succinate, artemisinin) and bio-fuels (e.g., ethanol, butanol, biodiesel etc.). Selected production routes must exhibit high yields, avoid thermodynamic barriers, bypass toxic intermediates and circumvent existing intellectual property restrictions. Historically, the incorporation of heterologous pathways relied largely on human intuition and literature review followed by experimentation [67,68]. Currently, rapidly expanding compilations of biotransformations such as KEGG [69] and BRENDA [70] are increasingly being prospected using search algorithms to identify biosynthetic routes to important product molecules. Several optimization and graph-based methods have been employed to computationally assemble novel biochemical routes from these sources. OptStrain [71] used a mixed-integer linear optimization representation to identify the minimal number of reactions to be added (i.e. knock-ins) into a genome-scale metabolic model to enable the production of the new molecule. However the combinatorial nature of the problem poses a significant challenge to the OptStrain methodology as the number of reaction database entries increase from a few to tens of thousands. At the expense of not enforcing stoichiometric balances, graph-based algorithms have inherently better-scaling properties for exhaustively identifying all min-path reaction entries that link a source with a target metabolite. Hatzimanikatis *et. al. *[72] introduced a graph-based heuristic approach (BNICE) to identify all possible biosynthetic routes from a given substrate to a target chemical by hypothesized enzymatic reaction rules. In addition, the BNICE framework was used to identify novel metabolic pathways for the synthesis of 3-hydroxypropionate in *E. coli *[73]. Based on a similar approach, a new scoring algorithm [74] was introduced to evaluate and compare novel pathways generated using enzyme-reaction rules. In addition, several techniques such as PathMiner [75], PathComp [76], Pathway Tools [77,78], MetaRoute [79], PathFinder [80] and UM-BBD Pathway Prediction System [81] have been used to search databases for bioconversion routes.

**Figure 5:**
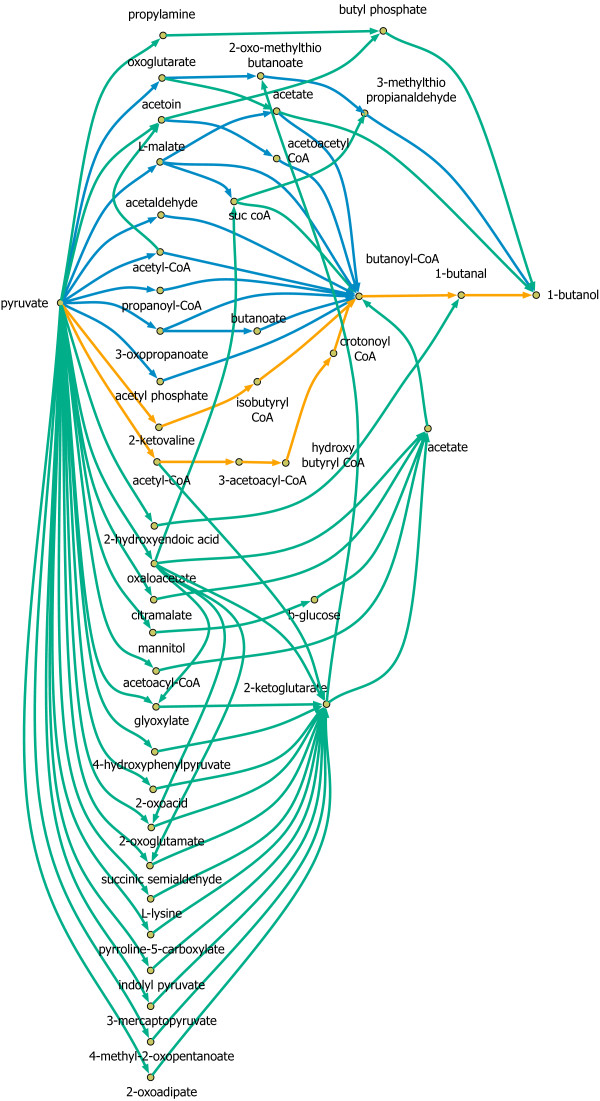
**Pathways from pyruvate to 1-butanol**. Using the MetRxn knowledgebase, we identified a large number of new pathways (green) as well as previously established ones (orange) and those identified found in a previous study [82] (blue).

We recently published [82] a graph-based algorithm that used reaction information from BRENDA and KEGG to exhaustively identify all connected paths from a source to a target metabolite using a customized min-path algorithm [83]. We first demonstrated the min-path procedure by identifying all synthesis routes for 1-butanol from pyruvate using a database of 9,921 reactions and 17,013 metabolites manually extracted from both BRENDA and KEGG. Here, we re-visited the same task using the full list of reactions and metabolites present in MetRxn to assess the discovery potential of using MetRxn. Figure 5 illustrates all identified pathways from pyruvate to 1-butanol before MetRxn (29, shown in blue) and the ones discovered after using MetRxn (112, shown in green). As many as 83 new avenues for 1-butanol production were revealed as a consequence of using the expanded and standardized MetRxn resource. In addition, the search algorithm recovered known [84-88] synthesis routes using *E. coli *for the production of 1-butanol (shown in orange). The first pathway involves the fermentative transformation of pyruvate and acetyl-CoA to 1-butanol using enzymes from *C. acetobutylicum *[89]. The second pathway uses ketoacid precursors [84]. This example demonstrates how the biotransformations stored in MetRxn can be used to traverse a multitude of production routes for targeted bioproducts.

## Conclusions

MetRxn enables the standardization, correction and utilization of rapidly growing metabolic information for over 76,000 metabolites participating in 72,000 reactions (including unresolved entries). The library of standardized and balanced reactions streamlines the process of reconstructing organism-specific metabolism and opens the way for identifying new paths for metabolic flux redirection. Moreover, the standardization of published genome-scale models enables the rapidly growing community of researchers who make use of metabolic information to understand metabolism at an organism-level and re-deploy it for various biotechnological objectives. By removing standardization and data heterogeneity bottlenecks the pace of knowledge creation and discovery from users of this resource will be accelerated. MetRxn is constructed in a way that allows for quick updating and tracking of changes that occur in the primary databases, as well as available parsing tools that allow for rapid import of new genome-scale metabolic models as they become available. By having exports in SBML, MetRxn's output can be directly interfaced with software packages such as the COBRA toolbox.

During the construction of the initial release of MetRxn, we managed to associate structures for over 8,800 metabolites and re-balanced more than 2,400 reaction instances across 44 metabolic models. This enables the genuine comparison of metabolic content between metabolic models. Preliminary results reinforce that that discrepancies between metabolic models echo not only genuine differences in metabolism but also assumptions and workflow followed by the model creator(s). Going forward, we will continue to expand MetRxn to include more genome-scale metabolic models and add additional tools to aid in their analysis. Because we anticipate that the scope and number of models will rapidly expand, we plan to invite and encourage the community to offer comments about metabolite and reaction information as well as provide feedback on MetRxn itself.

### Availability and requirements

MetRxn is available at http://metrxn.che.psu.edu. Its use is freely available for all non-commercial activity.

## Authors' contributions

AK generated the software and tools for MetRxn and assisted in drafting the manuscript. PFS participated in the design of the database, performed database curation and FBA analysis, and drafted the manuscript. CDM conceived the study, participated in the design of the database and edited the manuscript. All authors read and approved the final manuscript.
